# Potential application of spent mushroom compost (SMC) biochar as low-cost filtration media in heavy metal removal from abandoned mining water: a review

**DOI:** 10.1007/s13762-022-04617-7

**Published:** 2022-11-05

**Authors:** Z. Madzin, I. Zahidi, M. E. Raghunandan, A. Talei

**Affiliations:** Civil Engineering Discipline, School of Engineering, Monash University Malaysia, Subang Jaya, Malaysia

**Keywords:** Adsorption, Heavy metal, Biosorbent, Water treatment, Mushroom compost

## Abstract

Overpopulation and rapid development have put an increasing burden on the environment, leading to various water crisis. Importing water from abandoned mines as an alternative raw water source could be the next answer to alleviate water scarcity problems globally. However, due to its high heavy metals content, there is a need to find an economical and effective method to remove heavy metals before reusing it as potable water source. Biochar, a low-cost and carbon-rich biosorbent, has received increasing attention on its application as a remediating agent to remove heavy metals from water. Previous studies have revealed the potential properties of biochar as a heavy metal removal agent including high cation exchange capacity, high surface area, active surface functional groups, as well as efficient adsorption. Apparently, the most important factor influencing the sorption mechanism is the type of feedstock materials. Spent mushroom compost (SMC), a waste product from mushroom cultivation, has been found as an excellent biosorbent. SMC has received global attention as it is low cost and eco-friendly. It also has been proved as an efficient heavy metals remover from water. Nevertheless, its application as biochar is still scarce. Therefore, this review focuses on the potential of transforming SMC into modified biochar to remove heavy metals, especially from abandoned mining water. The present review emphasizes the current trends in adsorption methods for heavy metal removal from water, assembles data from previous studies on the feedstock of biosorbents to biochars, and discusses the potentials of SMC as a biochar for water treatment.

## Introduction

Access to clean water is essential for all living organisms to maintain well-balanced biological processes. However, increasing global water demand is due to rapid development and overpopulation, while massive disruption from industrial activities has resulted in various forms of pollution and water shortage. According to the United Nations, around 40% of the world’s population will suffer from water shortage by 2050 (Rahman et al. 2014). World Health Organization (WHO) and United Nations Children’s Fund (UNICEF) also reported that an estimation of 1.6 billion people will face lack access to safe household drinking water by 2040. Recently, due to COVID-19 pandemic, 3 out of 10 people faced lack of hygiene due to difficult access to clean water (Sahithya et al. 2022). Hence, to alleviate the water scarcity problems, one of the alternatives that has been implemented by some countries including Malaysia is by importing water from abandoned mining ponds as alternative water resources. However, the current standard operating procedures are not designed to remove high level of heavy metal contaminants in abandoned mining ponds, making such water is unsuitable as potable water. Thus, there is a significant need for a cost-effective, sustainable and practical solutions in removing high concentrations of heavy metals from ponds prior to clean water discharge.

Adsorption is a common method used in heavy metal treatment due to its effectiveness and economic advantage adsorbents (Fu and Wang 2011). This process has been approved to be the most promising technique in treating heavy metals other than the conventional method (Rosales et al. 2017). Many materials can be used as adsorbents to remove these heavy metals. However, the removal efficiency depends heavily on the physicochemical properties and the types of adsorbent. Hence, recent studies have been focused on searching the low-cost adsorbents with low maintenance, easy handling and high adsorption capacity properties. Low-cost adsorbents are distinguished for its scarcity process, abundant in nature, easily available, biomass waste products or waste materials from other industry (Abdolali et al. 2015). To date, numerous studies have demonstrated biochar as low-cost adsorbent to remove heavy metals from water treatments (Rosales et al. 2017). Biochar is a carbon-rich material made from biomass waste formed through thermal decomposition in limited oxygen condition. Biochar also is a great biosorbent in removing heavy metals from wastewater (Fu and Wang 2011; Wang et al. 2019; Yaashikaa et al. 2019) due to its large surface area and active functional groups that aids in metal adsorption. Compared to activated carbon, biochar is considered as less expensive and does not require any additional activation process. Therefore, biochar as an adsorbent filtration media in water treatment technology has gained more attention as it is economical, low maintenance but has high performance in removing heavy metals.

On the other hand, spent mushroom composts (SMC) are agricultural waste from mushroom crops harvested. According to Wu et al. (2019b), every 1 kg of mushroom production generates 5 kg of SMC residues. China covers 80% of the world’s mushroom production with more than 20 million tons of fresh mushrooms harvested. In Malaysia, approximately 100 tons of fresh mushroom harvested per annum, generating approximately 438 tons of spent mushroom composts left to be disposed (Phan and Sabaratnam 2012). However, as SMC contains high amount of organic contents (Frutos et al. 2016), many attentions have been given on its application as potential raw biomass for biosorbents and recently as biochar. In a previous study, SMC biochar was used as biosorbent to remove fluorine from water (Chen et al. 2016). Another study also has been done to explore the adsorption of SMC biochar to remove copper metal from aqueous solution (Jin et al. 2021). The findings revealed SMC biochar contains abundance of lignin and demonstrated effective copper removal.

However, there are limited studies on the application of spent mushroom compost (SMC) as the biochar filtration media for removing heavy metals especially in wastewater and stormwater treatment technologies as well as abandoned mining water. Therefore, the current review focuses on the potential of SMC as a low-cost and abundantly available material to remove heavy metal contaminants from water. In this study, the focus will be on the potential usage of SMC in water treatment. The discussion in this review is divided into three categories:

1. Abandoned mining water as an alternative water source and the conventional treatment technologies in removing heavy metals.
2. Converting biosorbents including SMC to potential biochar for heavy metal removals.
3. Potential applications of SMC biochar as filtration media for abandoned mining water.

## Abandoned mining water as an alternative water source

River is the primary raw water source in Malaysia, covering almost 97% of the demand, unlike countries that depend on groundwater and seawater. However, due to severe water pollution in rivers caused by rapid industrialization and urbanization in Malaysia, water scarcity has become an increasing concern. According to an assessment on lakes for meeting the water needs and demands in Malaysia, most of the lakes are polluted with crucial water quality parameters which were higher than the permitted levels set by the Department of Environment (DOE) Malaysia (Koki et al. 2018). Consequently, a significant project for alternative water sources has been implemented since 2007, where rainwater harvesting systems have been implemented in Selangor and Sarawak while other states like Kelantan, Perlis, Pahang and Terengganu use surface water and groundwater (Hamid 2015). Nevertheless, the water demand in Malaysia has an increasing trend and the water scarcity is still a problem. To address this issue, the authority of Selangor state has imported water from abandoned mining ponds as raw water resources (Kusin et al. 2016). However, this approach resulted in some disagreement among researchers on the water quality as abandoned mining ponds contain high concentrations of heavy metals (Lau et al. 2017). The World Health Organization (WHO) reported that most human diseases are from water sources, and one of them is from heavy metal contamination (Abdolali et al. 2017).

Anthropogenic and natural sources can contribute to high level of heavy metals, but researchers found that most water pollution cases are linked to human activities from rapid industrialization (Koki et al. 2018). The environmental impacts from pollution especially heavy metal contamination and their impacts on human have been discussed in many studies as presented in Table 1. This table shows that the heavy metals which are lead (Pb), manganese (Mn), zinc (Zn) and copper (Cu) are the most commonly found in the abandoned mining water and the impacts on human bodies from long term of exposure.

**Table 1:** Commonly observed heavy metals and their impacts on human bodies due to long-term exposure

Heavy metals	Sources	Symptoms	Health effects	References
Lead (Pb^2+^, oxidation state: ^+2^, ^+4^)	Application of lead in gasoline, drinking water, fuel combustion, paint industry, ceramic and dishware industry, PVC-mini blinds, solid waste combustion	Nausea, vomiting, thirst, diarrhoea/constipation, abdominal pain, load colic, lead palsy and lead encephalopathy	Anaemia, hypertension, kidney damage, miscarriage, disruption of the nervous system, irreversible learning impairment in infants and children, infertility, intellectual disorders, renal and hemopoietic system	Jan et al. (2015); Lim et al. (2012)
Copper	Mining, smelting, agricultural activities	Gastrointestinal distress	Anaemia, encephalopathy, hepatitis and nephritic syndrome, liver or kidney damage	Xu et al. (2019)
Zinc	Paint industry, plane industry, mining, smelting	Acute zinc poisoning, vomiting, diarrhoea, gastrointestinal symptoms	Anaemia, depression, lethargy, neurological signs, and thirsty	Azimi et al. (2017); Ding et al. (2016)

Water quality is assessed using many chemicals, physical and biological parameters set by authorities. In Malaysia, the National Water Quality Standards (NWQS) are formally used to develop the water quality index (WQI) and represent the overall status of a water source. The WQI uses the assessment recommended by the DOE in 1974 to evaluate the pollution levels in Malaysian rivers. In this process, six important water quality parameters including pH, ammoniacal nitrogen (AN), biological oxygen demand (BOD), chemical oxygen demand (COD), suspended solids (SS) and dissolved oxygen (DO) are calculated to classify the water into five classes starting from Class I (practically no treatment necessary) to Class V (not meant to be used at all) (DOE 2006). The details of these five classes are provided in Table 2. Consequently, due to the increasing speculations on the water quality status, numerous studies have contributed to the evaluation of the water quality in abandoned mining ponds throughout Malaysia.

**Table 2:** WQI classification for Malaysia (DOE, 2006)

Parameter	Unit	Class				
		I	II	III	IV	V
pH	–	> 7	6–7	5–6	< 5	> 5
Dissolved oxygen (DO)	mg/L	> 7	5–7	3–5	1–3	< 1
Biochemical oxygen demand (BOD)	mg/L	< 1	1–3	3–6	6–12	> 12
Chemical oxygen demand (COD)	mg/L	< 10	10–25	25–50	50–100	> 100
Suspended solids (SS)	mg/L	< 25	25–50	50–150	150–300	> 300
Ammoniacal nitrogen (AN)	mg/L	< 0.1	0.1–0.3	0.3–0.9	0.9–2.7	> 2.7
Water quality index (WQI)	–	< 92.7	76.5–92.7	51.9–76.5	31.0–51.9	> 31.0

Table 3 shows the heavy metals analyses in water from abandoned mines in Malaysia. Based on the data, there are a few abandoned mining sites that have high heavy metals concentrations that exceeded the standard limits for raw water quality by the Ministry of Health (MOH). Other than high concentrations of heavy metals, studies also have detected low pH in the abandoned mining ponds, indicating high level of heavy metals presented in the abandoned mine ponds, which was possibly related to acid mine drainage (AMD). AMD occurs due to the weathering of sulphide minerals, resulting in very acidic water and elevated concentrations of heavy metals (Kefeni et al. 2017). Hence, these studies concluded that there is an urgent need for appropriate water treatment before using the water as alternative raw water source.

**Table 3:** Heavy metal analysis in water from abandoned mining ponds in Malaysia

Location	State	pH	Heavy metals (mg/L)					References
			Cu	Mn	Pb	Zn	As	
Serendah and Biru Kundang lake	Selangor	5.7	0.06–0.1	ND	1.84	ND	ND	Hamzah et al. (2018)
Klang valley	Selangor	3.4–5.0	NA	> 0.028	6	NA	56	Koki et al. (2018)
Melaka	Melaka	3.4–5.0	NA	NA	6	NA	> 10	Koki et al. (2018)
Multiple locations	Perak	6.2–9.0	NA	NA	0.019–0.075	NA	0.004	Orji et al. (2013)
Multiple locations	Pahang	2.38–7.9	0.01–6	NA	0.54	0–9.86	0–0.02	Wan Yaacob et al. (2009)
Multiple locations	Pahang	5.0–7.99	0.01–0.025	0.104–1.299	ND	0.01–0.642	NA	Madzin et al. (2017)
Bestari Jaya	Selangor	3.2–7.2	0.003	0.647–0.727	0.001–004	0.013–0.239	0.01–0.18	Madzin et al. (2015)
Bukit Besi	Terengganu	2.5–6.5	0.001–0.19	2.03–7.82	0.003–0.009	0.021–0.166	< 0.001	Kusin et al. (2021)
MOH untreated raw water		5.5–9.0	1.0	0.2	0.1	5.0	0.05	Ministry of health

*ND* is not detected, *NA* not applicable

Generally, the water of abandoned mining ponds mostly contains heavy metal contaminants, leading to a great concern among researchers. Most researchers suggested that further treatment is required before releasing the treated raw water into river streams for water supply. However, there is no specific approach to treat heavy metals prior to release into water bodies. Conventional water treatment processes generally consist of series of steps. The primary treatment includes aeration, the secondary treatment consists of biological treatment to eliminate dissolved organic materials, and the tertiary treatment involves further improvement of the effluent quality. Heavy metal ions are typically treated in the third step of water treatment. Table 4 summarizes the conventional methods of heavy metal removals with respective advantages and limitations. The most appropriate and employed method among water treatment technologies is adsorption. Only certain heavy metals are successfully removed in the tertiary treatment, except for adsorption, which is known to remove high concentrations of heavy metals (Alias et al. 2020). Adsorption has already been applied as a supplementary treatment to remove organic and inorganic contaminants in wastewater (Vareda et al. 2019). The method is cheap, easy to operate and offers flexibility in design and operation (Nasir et al. 2019). The most common adsorbent used is activated carbon (Yang et al. 2019) due to its well-developed porous structure, high surface area and multifunctional groups. However, activated carbon is expensive and the recovery of heavy metals is a tedious process (Burakov et al. 2018). Therefore, more research and alternative technologies are required to remove heavy metal contamination in wastewater.

**Table 4:** Conventional methods in heavy metals removal from wastewater

Technology	Properties description	Advantages	Limitations	Effect on pH	Removal efficiency (%)	References
Chemical precipitation	Chemical reagent reacts with metal ions and precipitates as insoluble solid particles	Low capital cost Simple operation Safe operations	Sludge generation Slow metal precipitation Require massive chemicals to reduce metal ions	Optimal removal rate at pH 9–11. pH > 11 will cause dissolution of heavy metal compounds in precipitates and promotes the growth of metal hydroxyl complexes	80–99.7	Aziz et al. (2008; Barakat (2011); Lu and Chen (2018)
Ion exchange	Reversible interchange whereby insoluble substance (resin) removes ions from electrolytic solution and releases other ions of similar charge without any structural change in the resin	High treatment capacity High removal efficiency Fast kinetics	Not applicable in high metals concentrations Highly sensitive to pH Fouling of metal ions	Heavy metal removal works efficiently with pH	64–90	Abdullah et al. (2019); Agustiono et al. (2006); Fu and Wang (2011)
Coagulation- flocculation	Destabilization of cataloids (coagulation) Agglomeration of destabilized particles (flocculation)	Simple design; Low energy consumption High versatility	Toxicity of alum/polymeric coagulants; Sludge production Enable to remove heavy metals effectively	Optimal removal efficiency at pH 8–10 depending on the type of heavy metals; Zn^2+^ (pH = 8–10), Cu^2+^ (pH = 10)	95–99.9	Bora and Dutta (2019); Sun et al. (2019)
Membrane technology	Separation of substances across a semi-permeable material to pass through and excrete undesired substances	Small space requirement; low pressure; high separation; zero sludge production	Large capital investments Irreversible Membrane fouling Anti-hydrophobicity	Highest removal efficiency is obtained at pH ranging from 8–10	95–100	Abdullah et al. (2019); Agustiono et al. (2006); Fu and Wang (2011); O’Connor et al. (2018)
Adsorption	Flexible, simple and easy method where physical and chemical adsorption takes place	Low cost and maintenance Easy operation and handling Can be used to remove high concentrations of heavy metals	Production of waste products Performance and efficiency depend on the type of adsorbent Poor tolerant of different pH range	Very much dependent on the type of adsorbent	85–100	Abu Hasan et al. (2020); Alias et al. (2020)

## Converting biosorbents to biochar for heavy metals removal

Biosorbents are the most popular alternative adsorbents to replace the costly activated carbon as adsorption media. Biosorbents from biomass have drawn much attention due to biosorption mechanisms that can immobilize metals from industrial effluents (Niazi et al. 2016). Various biomass has been identified to produce low-cost biosorbents for water and wastewater treatments due to the ability in immobilizing heavy metals and feasibility to store more carbon, increase crop yields and enhance adsorption mechanisms (Li et al. 2017; Pan et al. 2019; Wang et al. 2019).

### Biomass waste as biosorbents

Producing low-cost biosorbents from agricultural wastes for removing organic and inorganic contaminants (including heavy metals) has been studied previously for variety of biomass materials such as sugarcane bagasse (Hussain and Qazi 2016; Mattos et al. 2015; Mohamed et al. 2017; Sarker et al. 2017), rice husk (Noor Syuhadah and Rohasliney 2012) and oil palm (Daneshfozoun et al. 2016; Mohd Salleh et al. 2018; Montoya-Suarez et al. 2016). Although these biosorbents showed satisfactory results in removing heavy metals through adsorption, the biosorbents have lower sorption capacity than other sorbents, such as activated carbon and ion-exchange resins. Hence, research has focused on enhancing the sorption capacity of biosorbents through chemical and physical modifications.

### Biomass waste as potential biochars

Biochars are chars produced from raw biomass via low oxygen thermochemical processes that increase the total surface area and produce strong active sites for adsorption (Mohanty et al. 2018; Phing et al. 2014). The entire process is similar to the production process for activated carbon, but activated carbon requires additional processes that need costly oxygen and strong acids for char activation (Mohanty et al. 2018). Biochars have received increasing attention due to their economic production and unique features, such as high carbon content, cation exchange capacity and large activation sites for metal binding (Wang et al. 2019). Various biomass materials have been transformed into biochars and critically investigated. The overall adsorption capacities in removing heavy metals are tabulated in Table 5, including SMC. The adsorption performance and efficiency of biochars are highly depending on the biochar properties.

**Table 5:** Biochar from various feedstock materials and the adsorption capacities in removing Cu(II), Zn(II) and Pb(II)

Biochar	Pyrolysis temperature (°C)	Residence time	Heavy metals	Adsorption capacity (mg/g)	References
*Agricultural and forest residues*					
Oil palm	700	12 h	Cu (II) Pb (II) Zn (II)	49.4 58.8 45.7	Samsuri et al. (2014)
Rice husk	700	12 h	Cu (II) Pb (II) Zn (II)	37.5 43.9 34.3	Samsuri et al. (2014)
Peat moss	800	90 min	Cu (II) Pb (II)	39.8 81.3	Lee et al. (2015)
Pistachio shell	550	1 h	Cu (II) Pb (II)	1.17 1.22	Komnitsas et al. (2015)
Peeled pine wood	700	3 h	Pb (II)	91.98	Komnitsas et al. (2015)
Hickory chips	600	2 h	Zn (II)	0.71	Ding et al. (2016)
Phyllostachys pubescens	450	3 h	Pb (II)	67.4	Zang et al. (2017)
Mushroom stick	800	90 min	Cu (II) Pb (II)	2.43 4.9	Chen et al. (2016)
Spartina alterniflora	500	2 h	Cu (II)	48.49	Li et al. (2017)
Nut shield	600	1 h	Pb (II)	4.61	Vítková et al. (2016)
Sugarcane bagasse	500	3 h	Cu (II)	86.96	Abdelhafez and Li (2016)
Orange peel	500	3 h	Cu (II)	27.86	Abdelhafez and Li (2016)
Cactus fibres	600	1 h	Cu (II)	3.5	Hadjittofi et al. (2014)
Porplyra tenera	500	1 h	Cu (II)	75.1	Kim et al. (2016)
E. Compresa (microalgae)	500	1 h	Cu (II)	137	Cho et al. (2015)
Colocasia esculenta	600	1 h	Cu (II)	2.31	Banerjee et al. (2016)
Swine and goat manure	800	1 h	Cu (II)	40.64	Zeng et al. (2018)
Pinewood	600	2 h	Pb (II)	4.91	Wang et al. (2019)
Plum stone	600	2 h	Pb (II)	47.05	Vítková et al. (2016)
Marine macroalgae	600	1 h	Cu (II) Zn (II)	23.16 22.22	Bakshi et al. (2018)
Sugarcane leaf	550	1 h	Pb (II)	103	Li et al. (2017)
Rice hull	400	2 h	Pb (II)	367.65	Han et al. (2016)
Hickory wood	600	1 h	Pb (II) Cu (II)	22.82 15.7	Wang et al. (2019)
Banana peels	230	1 h	Pb (II)	241	Zhou et al. (2017)
SMC	500	3 h	Cu (II) Zn (II) Pb (II)	364.2 333.2 564	Abdallah et al. (2019)
SMC	300–600	4 h	Cu(II)	65.6	Jin et al. (2021)
*Industrial by-products*					
Sewage sludge	900	20 min	Cu (II) Pb (II) Zn (II)	0.19 0.926 0.2	Chen et al. (2014)
Dried sewage sludge	650	30 min	Pb (II) Cu (II)	40.30 6.70	Otero et al. (2009)
Anaerobically digested sugarcane bagasse	600		Pb (II)	135.5	
Anaerobically digested whole sugar beet	600		Pb (II)	40.8	
Anaerobically digested animal waste	600		Pb (II)	51.4	Inyang et al. (2012)

From the table, it can be concluded that these feedstock materials that have been transformed into biochars have very high potential in removing heavy metals. However, different types of biomass biochar give different adsorption capacities. Thus, it is crucial to explore different types of biomass and investigate their feasibility to remove heavy metals, as well as the performance of SMC as biochar in heavy metal removal. Hence, more research should be done in the future to utilize the feasibility of SMC biochar especially as filter media in bioretention system.

### 3.3. Spent mushroom compost as biosorbent

Mushroom production and demand in Malaysia have been gradually increasing, and mushroom is identified as one of the high-value commodities under the National Agro-food Policy (2011 − 2020). Mushrooms are delicious, highly nutritional, low in calories, free from cholesterol and contain proteins, vitamins, fibre and also rich in potassium, iron and phosphorus minerals (Mattos-Shipley et al. 2016). The demand for mushrooms increases yearly, and improper handling of SMC may result in environmental pollution. SMC contains a mixture of cellulose, hemicellulose and lignin, which contain high amount of hydroxyl, carboxyl, carbonyl, amino and phosphate that act as active functional groups on the surface of this material. These active functional groups favour metallic ion biosorption to achieve high adsorption capacity (Xiao-jing et al. 2014). SMC has been applied extensively in environmental remediation treatments. In many studies, SMC has been used as biosorbents to remove organic and inorganic pollutants. Applications of SMC as biosorbent to remove heavy metals are presented in Table 6. Generally, SMC has shown great adsorption capacity in removing single and multiple metal ions and is an excellent biosorbent as it minimizes the plugging in bioreactors and has large pore spaces and small void volume (Muhammad et al. 2017). On the other hand, SMC contains different types of polymers such as lignin, cellulose and hemicellulos which are degraded into numerous pores that are suitable for metal adsorption (Kulshreshtha 2019).

**Table 6:** Application of SMC as biosorbent to remove heavy metals from literature

Biosorbent	Heavy metals	Adsorption capacity (mg/g)	Removal efficiency (%)	References
Spent mushroom compost (SMC)	Cr(VII)	9.327	80–98	Dong et al. (2018)
	Pb (II)	149.1		Liu et al. (2018)
	Mn (II)	17.25		Kamarudzaman et al. (2015)
	Cu (II)	50		Xiao-jing et al. (2014)
	Cd(II) Pb(II) Fe(II) Co(II) Mn(II) Zn(II) Cu(II)			Corral-Bobadilla et al. (2019)
	Pb(II)		60–97.87	Molahid et al. (2018)
	Mn (II)	3.341		Kamarudzaman et al. (2015)
	Cd(II) Pb(II) Cu(II)	40.43 15.16 36.2		Frutos et al. (2016)
	Ni(II)	3.04		Tay et al. (2011)

However, one of the limitations in utilizing SMC is the compost which can easily get exhausted, affecting its long-term performance (Roychowdhury et al. 2015). Thus, to improve the biosorption capacity of SMC, chemical or physical modification is introduced to pre-treat SMC biosorbents. However, very limited studies have been done. For example, the co-pyrolysis of SMC and macroalgae improved the cationic dye adsorption capacity by 2.2 times higher than the raw SMC (Sewu et al. 2017). The study added that more oxygen-containing groups, exchangeable cations and coarser surface morphology were obtained through this technique, improving adsorption synergism. Meanwhile, SMC adsorbent was modified using cationic surfactants (Zang et al. 2017) and the uptake capacity of Cr (VI) improved to 21.44–27.34% using chemically modified SMC with cetyltrimethylammonium bromide (CTAB). These studies showed that the modification of SMC is effective in enhancing the adsorption capacity. However, further studies on SMC modification need to be conducted, specifically on physical modifications where simple operation, easy handling and minimal investment are required.

### Role of spent mushroom compost as biochar

The application of SMC as biochar is still relatively new in the research field. Therefore, the discovery of SMC as biochar is limited and needs to be explored thoroughly. Most studies of SMC biochar are pioneered by the countries producing mushrooms on a large scale, where the waste production has become a waste management problem and adequate management is required to turn the waste into a potential feedstock especially for biochar. Table 7 lists the applications of SMC biochar to remove heavy metals from previous literatures. Abdallah et al. (2019) studied the role of SMC biochar in removing heavy metals (Zn, Cu and Pb) from wastewater in both batch and continuous systems. In this study, SMC biochar was produced via slow pyrolysis and tested to remove heavy metals by considering the effects of several factors including pH, initial solution, contact time, temperature and competitive adsorption. The characterization of SMC biochar showed the presence of functional groups and the value of cation exchange capacity. Based on the results, the maximum adsorption obtained was 564 mg/g for Pb, 364 mg/g for Cu and 332 mg/g for Zn, higher than the adsorption for biochars from apricot stone, soybean hulls and pecan shells. The adsorption efficiency of SMC biochar was evaluated to remove Pb (II) (Wu et al. 2019). The authors mentioned that the physicochemical properties of the SMC biochar (i.e. large Brunauer–Emmett–Teller (BET) surface area, small pore structure and abundant functional groups) contributed to the high adsorption capacity of Pb (II) with the maximum adsorption capacity of 326 mg/g.

**Table 7:** Application of SMC biochar in removing heavy metals from aqueous solution to date

Biochar	Heavy metals	system	Initial concentration (mg/L)	Adsorption capacity (mg/g)	Description	References
Spent mushroom compost	Pb Cu Zn	Batch and column	30	Pb 564 Cu 364 Zn 332	Biochar was prepared by carbonization and evaluated by several laboratory factors including initial pH, contact time, temperature and competitive adsorption in removing mixed metals	Abdallah et al. (2019)
Spent *P.ostreatus* substrate	Pb	Batch	1000	326	Two types of SMC were chosen as biochar raw materials to investigate Pb adsorption performance and study the physicochemical properties	Wu et al. (2019a)
Spent shiitake substrate	Pb	Batch	1000	398		Wu et al. (2019a)
Spent mushroom compost	Cu	Batch and column	50	52.6–65.6	16 biochars were produced from four types of SMC to study the effect of pyrolysis temperature and characterization in removing Cu(II) in batch and continuous system	Jin et al. (2021)
Spent mushroom compost	Cu Zn Cd	batch	100	68.1 55.2 64.8	Novel application of mineral-rich biochar from SMC with various pyrolysis temperature (350–750 °C) to remove mixed metals	Agaricus (2021)
Spent mushroom compost	Pb Cd	batch	700	262.75 75.82	SMC biochar to remove Pb and Cd from water with effect of pyrolysis temperature	Chang et al. (2020)

Jin et al. (2021) conducted an advanced study on the characterization of various species of SMC and evaluated the adsorption performance in removing Cu (II) from aqueous solution. A total of 16 biochars from four types of SMC were utilized and produced at different pyrolysis temperatures. All SMC biochars showed highly different properties and were significantly affected by the pyrolysis temperatures. Furthermore, all four SMC biochars showed effective removal of Cu (II) with the maximum adsorption capacities between 52.6 and 65.6 mg/g for biochars pyrolyzed at 600 °C. These properties define each unique condition. Different materials show different performances and efficiencies, depending on their physical properties (i.e. surface morphology and pore size), chemical composition and materials. The performance of biochars as adsorbents is also influenced by pH, initial metal concentration that involves adsorption isotherms, contact time (adsorption kinetics) and adsorbent dosage.

Other than that, the production of biochar using different pyrolysis temperature affects the overall removal performance of SMC biochar. Chang et al. (2020) and Agaricus (2021) studied the effect of pyrolysis temperature on the adsorption capacities and revealed SMC had the highest removal at high pyrolysis temperature (> 650 °C) due to rapid increase in surface area and well development of mesoporous structure causing effective adsorption of heavy metals.

The performance of adsorbents in various field applications is strongly dependent on its characteristics. The physical and chemical characteristics vary significantly depending on the raw material and the production processes (Spokas et al. 2014).

Physical characteristic such as surface morphological and pore size is a crucial property to measure adsorption efficiency. Chemical properties also contribute to the adsorption performance of an adsorbent. Table 8 shows the comparison of physicochemical properties of SMC as biosorbent and biochar at 700 °C as compared to an activated carbon from previous studies. This information compiles a thorough explanation on how SMC biochar has been a great improvement from its raw material which can outstand or has similar performance of activated carbon. Surface area and pore volume of biochars are relatively important features affecting adsorption and retention properties of the materials. Masebinu (2019) found that the uptake of adsorbate into biochar relies on the accessible volume of micropores and surface area of the biochar. Based on the radius of the openings, three types of pores can be defined: (1) micropores, which are responsible for the surface area and immersive adsorption capacity factor of biochar; (2) mesopores, which are critical for liquid–solid adsorption processes; and (3) macropores, which are responsible for aeration, hydrology and bulk soil structure (Qambrani et al. 2017).

**Table 8:** Comparison of physicochemical properties of SMC biosorbent and SMC biochar

Media	pH	BET surface area (m^3^/g)	Pore volume (cm^3^/g)	Pore size (nm)_	Elemental analysis				References
					C	H	N	O	
SMC biosorbent*	7.2	0.32	0.003	30.73	54.3	3.45	2.16	24.9	Corral-Bobadilla et al. (2019)
SMC biochar at 700 °C*	12.07	218.70	0.138	2.52	44.85	1.23	1.09	9.55	Wu et al. (2019)
Activated carbon*	5.84	1162	0.6193	10.6	41.59	6.18	1.67	45.98	Tsai et al. (2020)

*Values may vary

**Fig. 1:**
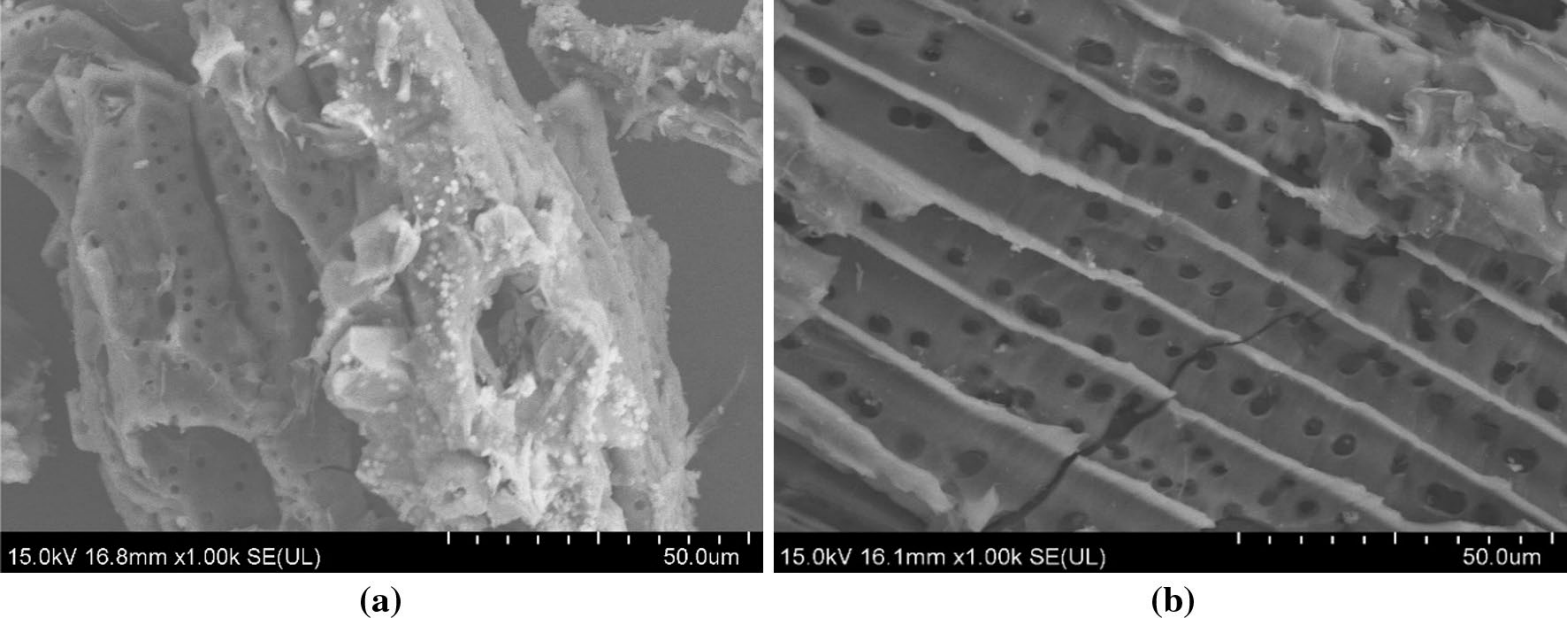
SEM images of **a** SMC biosorbent and **b** SMC biochar

As stated by Chen et al. (2016), greater pore volume and Brunauer–Emmett–Teller (BET) analysis grants stronger physical adsorption capacity, whereby BET is a method to calculate the surface area involving nitrogen adsorption (Shaheen et al. 2018). From the table, the BET surface area of biochar with pyrolysis temperature of 700 °C is 200 times higher than the SMC as raw materials. The pyrolysis process decomposed the lignin material, released the volatile substance and enhanced the surface area of biochar. High surface area contains more micropores which are responsible for adsorption of heavy metals. Hence, higher pore volume showed by SMC biochar compared to biosorbent aids in adsorption and increases the adsorption performance. Although high surface area gives an excellent adsorption performance, smaller pore size is needed to increase the adsorption rate and this is shown with the smaller size distribution of SMC biochar compared to biosorbent. Kizito et al. (2015) concluded that smaller particle size increases the adsorption rate due to shorter diffusion path and causes higher penetration of the adsorbate into the pores of the adsorbent, increasing adsorption performance. Further statement on the porosity can be supported by the surface morphological images using scanning electron microscope (SEM) in Fig. 1. From the SEM images, it is shown that the porous image of SMC biosorbent is enhanced by the pyrolysis process which promotes the formation of micropores in SMC biochar.

Chemical properties such as pH and elemental analysis play significant roles in the adsorption of heavy metals. SMC biochar has slightly higher pH than SMC biosorbent and this favours the adsorption of heavy metals. High pH contributes to better heavy metals adsorption by biochar (Mohan et al. 2014). Samsuri et al. (2014) found that low pH creates higher hydrogen ion condition which competes with heavy metals for the sorption sites. High pH adsorbent can also act as alkalinity generator to increase the acidic pH in acid mine drainage, which subsequently facilitates the condition for metal removal (Muhammad et al. 2017). Hence, by an increase in pH, more adsorption sites are available for heavy metals. Additionally, elemental analysis is an important parameter as it exhibits the elemental composition of feedstocks and different materials have different proportions of element composition, thus exhibiting different properties and adsorption capacities. The content of carbon in the biochar is important as it gives idea about the stability of the biochar. The carbon content in SMC biochar is highly similar to activated carbon hence explaining the stability of the biochar.

### Perspectives

In summary, the role of SMC as biochar has been proven effective in removing heavy metal from aqueous solutions. Previous studies have assessed the applications of SMC biochar in removing various heavy metals in a batch and column experiments and concluded the potential of SMC biochar as an effective biosorbent. SMC biochar is not just highly abundant, and it is also economically practical as it reduces disposal cost and promotes sustainability. Additionally, this carbon-rich material has its unique features such as large surface area that promotes higher adsorbent efficiency, just like other feedstock materials and activated carbon. Therefore, further application to utilize SMC biochar is recommended especially as filtration media which has not been explored yet.

## Application of biochar as filtration media 

As mentioned in Introduction, there is no study yet on incorporating SMC biochar as a filter media to treat heavy metals related to mining water. Most of previous studies were done in a batch and column experiments using other biochars and the potential application of these biochars to treat acid mine drainage was explored. In a study by Oh and Yoon (2013), poultry litter-derived biochar was used to treat heavy metal contaminations from acid mine drainage. The study used the poultry litter-derived biochar produced from slow pyrolysis at 400 °C in both batch and column experiments. The study revealed that biochar could treat heavy metals at low pH with metals removal of 99%, 61%, and 31% for Zn, Mn, and SO4, respectively. The authors reported that the biochar reacts as an alkaline generator, increasing the pH using the existing carbonate minerals from the biochar. Most of the heavy metal removal mechanisms include precipitation and sorption on the biochar surface. Similarly, microbially enriched poultry litter-derived biochar was applied to decontaminate mine drainage (Soares et al. 2018). The cow manure sulphate-reducing bacteria (SRB)-enriched biochar was used as the remediating media to treat sulphate from mining water. The biochar reduced 41% of sulphate concentration and was 39% more effective than other treatment methods. The high surface area of the biochar assists the overall removal of sulphate. Interestingly, the presence of SRB in different environments showed potential in treating heavy metals. However, by using the SRB-rich biochar, nutrient leaching might occur, causing competitive interactions and consequently leading to poor adsorption.

The conventional filter media using soil layer can provide successful physical–chemical and biological treatments. However, the performance was variable and research proved that it has first flush effect and transient wetting and drying which could hinder contaminant attenuation as well as re-immobilizes contaminants which leads to advanced design using other materials that is readily available, replaceable and inexpensive which includes biochar (Tsang et al. 2019). On the other hand, the application of biochar as filter media for stormwater treatment has been religiously explored and shown imperative result in removing contaminants. Selection of an optimum filtration media is crucial as it will influence the heavy metal removal. The roles of biochar as filter media in the best management practices (BMPs) include bioretention systems (Biswal et al. 2022), low impact development (LID) (Mohanty et al. 2018), stormwater biofilters (Valenca et al. 2021) and anaerobic bioreactors (Küçükağa et al. 2022). Two common properties for filtration media are (1) high hydraulic conductivity to minimize flooding and (2) high storage volume to enhance contaminant removals. Hence medium–coarse sand is commonly used to maintain high conductivity. While clay is used to increase the storage volume, it lowers the hydraulic conductivity. In contrast, biochar provides both advantages simultaneously. Biochar has unique property of extensive internal pore structures which not only increase storage volume but also increase hydraulic conductivity (Mohanty et al. 2018). Thus, to alleviate the efficiency of stormwater treatment facilities, researchers have attempted to enhance the performance by mixing it with suitable materials including biochar.

Biochar as filter media exhibits outstanding performance in removing heavy metals. A study using wood-derived biochar for stormwater treatment using bioretention column demonstrated highly efficient metal removal and supressed desultory degree of metal remobilization with metal removal of 50–70% (Sun et al. 2020). Sun et al. (2020) mentioned that biochar could replace activated carbon (AC) in removing contaminants. While it cost much lesser than AC, its outstanding metal-binding adsorption effectively eliminates heavy metals and its mechanism of action is diverse due to its large surface area and multiple active functional groups. Moreover, Tsang et al. (2019) published a review paper on the novel application of biochar in stormwater harvesting and gathered various heavy metals removal efficiencies using various biochar types. The study added, to enhance biochar adsorption performance, higher bed height can lengthen the life span of adsorbent while increasing flow rate and metal concentration would fasten the exhaustion rate. Hence, column design and filter media play important roles in influencing the adsorption performance. However, competition for binding sites of multiple elements could raise a challenge where a study showed a significant drop of Zn in multiple metal solutions compared to Cd and Cu (Park et al. 2016). Interestingly, biochar-amended woodchip bioreactor to treat heavy metals in stormwater was evaluated in a pilot scale and was found capable of removing nitrate and five metals (Ashoori et al. 2019).

Additionally, Biswal et al. (2022) reviewed biochar-based bioretention systems for removal of chemical and microbial pollutants including heavy metals. The authors highlighted that biochars have been successfully used as additional adsorptive media to the existing filter media in order to improve heavy metal removals. The authors also reported that the dominant removal mechanisms in such systems are sorption and ion exchange.

### Heavy metal removal mechanisms of biochar

Interestingly, adsorption is not the only removal mechanism in heavy metals removal, although it is known as the most important mechanism. There are many other mechanisms controlling the heavy metals removal in water treatment system (e.g. surface micro-precipitation, ion exchange, electrostatic attraction and chemical complexation). Adsorption is mainly associated as the main removal method as it may have a high sorption capacity for metallic contaminants. Mandu et al. (2015) reported that biochars have an enormous surface area with an astounding pore network, consisting of micropores, mesopores and macropores. Besides, the biochar removal capacity is influenced by its surface conditions, feedstock materials and pyrolysis status. Biochars with high surface areas and pore volume have strong interaction with metallic ions because the ions can be physically adsorbed onto the surface of biochars and remained in the pores. Heavy metal ions in the aqueous solution diffuse from the solution onto the surface of biochars with an opposite surface charge. Hence, the metal ions attach to the surface and are removed from the solution. This type of adsorption occurring at the surface of biochars is associated with van der Waals forces (Sulyman et al. 2017). This method does not involve any chemical bonds. Therefore, surface area and porosity are essential for biochars.

Apart from adsorption, ion exchange with dissolved metal species is argued as the most dominant mechanism (Fakhre and Ibrahim 2018). The ion-exchange mechanism involves the exchange of ionizable cations on the surface of biochars with metallic ion contaminants. Several researchers suggested that the abundance of negatively charged sites on the surface of biochars provided by multiple functional groups, such as carboxyl, hydroxyl and phenol (–COO− and –OH−) bind the metallic ions, such as Cu, Pb and Zn (Joseph et al. 2019; Shaheen et al. 2018; Shamsollahi and Partovinia 2019). Dai et al. (2018) explained the ion-exchange mechanism and proposed that the removal of heavy metals is accelerated by carboxyl and hydroxyl groups, as well as electron donor functional groups (–C–OH, C–O and C–O–R) that promote the chemisorption of Cu^2+^, Pb^2+^ and Zn^2+^ on the surface of biochar. The pH of the aqueous solution is vital in this mechanism. Functional groups (phenol) can deprotonate metal ions by a decrease in pH according to the following chemical formula in (1):

1 $$Me^{2 + } + 2\left( { - ROH} \right) = Me\left( {RO} \right)_{2} + 2H^{2 + }$$

Meanwhile, a study observed that the most optimum deprotonation at higher pH (3 ≤ pH ≤ 5) and an increase in negative charges allow heavy metals to coordinate with the surface functional groups and increase the removal efficiency (Paranavithana et al. 2016). This statement is supported by Kaya and Ozer (2014), where adsorption decreased with a decrease in pH.

During sorption, the formation of solids in a solution or on the surface of the adsorbent is inevitable due to the binding of metallic ions and the presence of chemical groups, hence creating precipitation. This mechanism is often correlated to the immobilization of heavy metals by biochars. Moreover, precipitation is favoured with strong interaction between metallic ions and plant biochars. The biochars produced at high temperatures will have high mineral matter content (Ca, Mg, Fe, Cu and Si). Hence, this mineral matter will come in contact with metallic ions and successfully immobilize the materials through precipitation (Shen et al. 2019).

Many biochar applications have only been conducted in batch and column experiments. In SMC biochar, the alkalinity of the biochar promotes precipitation, which could be proven in the X-ray diffraction patterns where the peak intensities show a crystallization process in the form of quartz. The functional groups present in SMC biochar are mainly aromatic and aliphatic functional groups that provide *π*-electrons, thus promoting adsorption. This property could be proven by the images from Fourier transform infrared (FTIR) characterization before and after adsorption as the peaks stretched and changed (Wu et al. 2019). Removal mechanisms can be best explained in the adsorption kinetics test. For example, in the batch experiment, the high R2 values in the pseudo-second-order model for the kinetics test indicate the involvement of chemisorption (Abdallah et al. 2019). Jin et al. (2020) determined the dominant adsorption mechanism for four types of SMC biochars through precipitation, followed by π-complexation. The complexation mechanisms involved surface functional groups abundant in SMC biochars, highlighting a promising low-cost adsorbent for heavy metal removal. Therefore, it is crucial to further explore the potential of these biochars and to identify the maximum adsorption efficiencies for an optimum performance of metal removal.

### Perspectives 

Although biochars have been applied as sorbents in removing organic and inorganic contaminants, little attempt has been made to employ biochars as a reactive media associated with mining water. Based on a review study on SMC biochar, theoretically, SMC biochar is an economical and effective approach to be used as filtration media to remove heavy metals from mining water. The high pH characteristic of SMC biochar will be a great alkaline generator to treat the low pH mining water. Its many physical and chemical composition features will provide multiple binding sites and enhance the adsorption performance. Therefore, further studies on SMC biochar as a potential filter media in bioretention systems could be essential, specifically for applications such as mining water treatment. It is also suggested that further study on the adsorption efficiencies, the long-term performance and the new applications of SMC biochar in large field-scale projects need to be carried out.

## Conclusion

A review on biochars and the remediation of heavy metals from water bodies using biochars have been presented in this paper. As demand on clean water is escalating, global water scarcity has become a vital issue. Therefore, importing water from abandoned mines as an alternative raw water source could be a feasible option to alleviate the global water scarcity problem. However, heavy metals contamination in mining water is also an issue need to be addressed before the water can be reused, and the removal of heavy metals can be costly. Thus, there is also a need to find an economical and effective method to remove heavy metals prior to further use. Biochar is a carbon-rich material with large surface area and active functional groups that has higher adsorption efficiency than the conventional biosorbents. This economical biochar improves heavy metal removal via the adsorption mechanism, which can be a promising technology in the future. The effectiveness of biochar depends largely on the feedstock material and the water quality conditions which include pH, initial metal concentration, contact time and adsorbent dosage. This review has evaluated the potential application of SMC that offers many unique features as biosorbents, demonstrating effective heavy metal removal. The limitations of SMC as biosorbents have been reviewed and further development has been conducted to physically modified SMC into biochar.

The role of SMC as biochar has been critically assessed and reviewed. The properties of SMC biochar with larger surface area, more active functional groups and the ability to remove heavy metals have been discussed extensively, and the comparison of physicochemical properties of SMC as biosorbent and biochar has been highlighted in this review. These conditions affect the overall performance of heavy metal removal and the immobilization of heavy metals on the biochar. This review has also examined the overall heavy metal removal mechanisms, which are the critical factors for biochars from various feedstock materials, including SMC, to clearly explain the processes occurring in aqueous solution. Adsorption and ion exchange are the most common removal mechanisms, along with the interactions in electrostatic forces and precipitation. The efficiencies of these mechanisms have been further described in terms of kinetics and adsorption isotherms for explaining the mechanics of the process. In short, biochars are feasible, cost-effective materials and effective in removing heavy metals from aqueous solutions, with the potential to remove heavy metals in mine drainage treatment. Biochars are readily available in large quantities and can simultaneously reduce the disposal costs and promote sustainability.

Therefore, future research should be conducted to explore the role of SMC biochar and evaluate their application on a pilot scale. Although many researchers have considered the production of biochar from numerous waste materials to remove heavy metals, the application of biochar in removing mining water is still lacking. To date, no study has been conducted on the performance of mushroom compost as biochar in removing heavy metal contaminants in mine drainage treatment. This information is highly important for the characteristics of mine drainage treatment containing high heavy metal contents and sometimes can have low pH, thus affecting the overall performance of biochar. The future approach of batch and continuous laboratory-scale experiments is therefore recommended. Pilot tests can be conducted to evaluate the overall adsorption performance, the kinetics and adsorption isotherms and the optimization study. In conclusion, there is a need to determine whether SMC biochar can remove heavy metals in abandoned mining water.
